# Nasal *Staphylococcus aureus* and Methicillin-Resistant *S*. *aureus* Carriage among Janitors Working in Hospitals in Northern Taiwan

**DOI:** 10.1371/journal.pone.0138971

**Published:** 2015-09-25

**Authors:** Chun-Jui Chang, Ning-Chun Chen, Chong-Kei Lao, Yhu-Chering Huang

**Affiliations:** 1 Department of Medicine, College of Medicine, Chang Gung University, Kweishan, Taoyuan, Taiwan; 2 Department of Pediatrics, Chang Gung Memorial Hospital at Linko, Kweishan, Taoyuan, Taiwan; University of Minnesota, UNITED STATES

## Abstract

**Background:**

*Staphylococcus aureus* is an important cause of infection, and brings additional concern with methicillin resistance. In addition, nasal methicillin-resistant *Staphylococcus aureus* (MRSA) colonization rates among health care workers are higher than that for general population. To determine the prevalence rate and risk factors for the colonization of *S*. *aureus*, *including* MRSA, among janitors working in hospitals in northern Taiwan, we conducted this study.

**Methods:**

Between June and August, 2014, a total of 186 janitors, 111 working in hospitals and 75 working in non-medical institutions, were recruited. Specimens were obtained from the nares of the subjects for the detection of *S*. *aureus*, with a questionnaire completed for each subject. All the *S*. *aureus* isolates, including MRSA and methicillin-susceptible *S*. *aureus* (MSSA), were further molecularly characterized.

**Results:**

The nasal carriage rate of *S*. *aureus* was 15.3% for hospital janitors and 13.3% for non-medical janitors. The carriage rate of MRSA was 3.6% for hospital janitors and 1.3% for non-medical janitors. No statistically significant difference was found in the nasal carriage rate of *S*. *aureus* (p = 0.707) and MRSA (p = 0.65) between hospital janitors and non-medical janitors. Hospital janitors working in hospital more than 6 years and cleaning microbiologic laboratories were significantly associated with nasal *S*. *aureus* colonization. All 5 MRSA isolates carried either staphylococcal cassette chromosome type IV or V and three of them belonged to sequence type (ST) 59, the community clone prevailing in Taiwan. Of the 22 MSSA isolates, six pulsotypes were identified, with one major type for 14 isolates (shared by five STs) and another type for 4 isolates (all belonged to ST 188).

**Conclusion:**

Exposure to the hospital environment may not increase the nasal carriage rate of *S*. *aureus*, including MRSA, among janitors in hospitals in Taiwan. However, for janitors in the hospital setting, working for more than six years in hospital and cleaning laboratories may be risks factors for carrying *S*. *aureus*.

## Introduction

*Staphylococcus aureus* is an important cause of skin and soft-tissue infections (SSTIs), endovascular infections, bacteremia, and sepsis in both hospitals and communities, and becomes increasingly resistant to methicillin. Methicillin-resistant *S*. *aureus* (MRSA) isolates were once confined largely to hospitals, other health care environments, and patients frequenting these facilities [1]. Since 1990s, MRSA were epidemiologically categorized into two groups, namely healthcare-associated (HA-MRSA) and community-associated (CA-MRSA), depending on the patients with or without risk factors, including recent hospital admission or operation history, long term accommodation in health care facilities, indwelling catheter or hemodialysis.

Initially, there are great differences in the molecular characteristics between CA-MRSA and HA-MRSA, eg. the former usually carried type IV or V staphylococcal cassette chromosome *mec* (SCCmec), had limited antibiotic resistance, and possessed Panton-Valentine leucocidin (PVL) genes, while the latter tended to exhibit multiple drug resistance, carry SCCmec II, III, but did not carry PVL genes [2–4]. However, various MRSA clones have spread between communities and hospitals, particularly CA-MRSA transmitted in hospital settings, making the blurred distinction between CA-MRSA and HA-MRSA [5–7].

*S*. *aureus* can colonize on anywhere of human bodies, particularly in the anterior nares. The carriage of *S*. *aureus*, including MRSA, is well known to be a significant risk factor for subsequent infection. In Taiwan, nasal MRSA colonization rates among health care workers (HCWs) (5.0–7.8%) [8] and pediatricians (6.8%) [9] were significantly higher than that for general population (3.8%) [3, 8, 9]. The role of janitors working in hospitals is of great importance. In direct contact with biological hazards, it may pose a potential threat to the health issue of the janitors themselves and individuals related with them. However, the colonization rate of *S*. *aureus* and MRSA among hospital janitors, who are exposed to the same environment as HCWs in hospitals in Taiwan, has not been reported. This study was conducted to determine the prevalence rate and risk factors for the colonization of *S*. *aureus* and MRSA among janitors working in hospitals in Taiwan by comparing those working non-medical institutions. Once identified, the molecular characteristics of *S*. *aureus* isolates from these janitors were determined and compared.

## Materials and Methods

### Ethic statement

This study was approved by the institutional Review Board of Chang Gung Memorial Hospital and a written informed consent was obtained from each subject before each survey for nasal carriage of MRSA.

### Subjects

Between June and August, 2014, janitors working in two hospitals (Chang Gung Memorial Hospital at Linkou and Taoyuan, respectively) and working in 13 non-medical institutions were invited for survey of nasal carriage of MRSA. The 13 non-medical institutions included nine universities (Chang Gung University, Chang Gung University of Science and Technology, National Taiwan Sport University, National Taipei University of Education, National Taipei University, National Taiwan Normal University, Taipei Medical University, Soochow University and National Taipei University of Nursing and Health Science) and three department stores (Tonlin Department Store Co., Ltd., Far Eastern Department Stores Co. Ltd. and Shin Kong Mitsukoshi Department Store Co., Ltd.) located in Taoyuan city and Taoyuan city Government. Eligible subjects were invited to be sampled from their anterior nares for detection of *S*. *aureus* nasal carriage after a written consent was obtained, and to complete a questionnaire regarding the risk factors for MRSA acquisition.

### Microbiologic methods

A nasal swab specimen was collected from both anterior nares of each subject by a dry sterile swab. Each swab was rubbed against the anterior 1 cm of the nasal vestibular wall of both nares. Then the swab was placed into the agar gel transport medium (Venturi Transystem, Copan Innovation Ltd, Limerick, Ireland) and sent to the laboratory for culture. There, swab specimens were streaked on 5% blood agar plates (BD Diagnostics, Sparks, MD) and incubated at 37 degree Celsius overnight. Isolates that presented with β-hemolysis and a coagulase-positive reaction were identified as *S*. *aureus*. MRSA and methicillin-susceptible *S*. *aureus* (MSSA) identification was done by the disk diffusion method, using cefoxitin disks (potency 30 mg) to determine the antibiotic susceptibility, in accordance with the Clinical and Laboratory Standards Institute guidelines [10].

### Antimicrobial susceptibility testing

The susceptibility of MRSA isolates to ten antibiotics including vancomycin, teicoplanin, linezolid, doxycycline, penicillin, ciprofloxacin, erythromycin, fusidic acid, clindamycin, trimethoprim-sulfamethoxazole was determined by the disk-diffusion method according to the standards of Clinical and Laboratory Standards Institute [10].

### Molecular characterization

For each *S*. *aureus* (both MRSA and MSSA) isolates, chromosomal DNAs were extracted for molecular characterization by pulsed-field gel electrophoresis (PFGE) with *Sma*I digestion [11], SCC*mec* typing (for MRSA only [12]), presence of Panton-Valentine leukocidin (PVL) genes [13], multilocus sequence typing (MLST) [14] and spa typing [15]. These methods were described in details anywhere previously [15–18].

### Questionnaire

Each subject was requested to complete a questionnaire including demographics, work experience and job details, and any possible risk factors for the colonization of MRSA. Demographic data included age, gender, education level, race, family members especially children, smoking, alcohol and betel nut habits. Any chronic underlying disease, use of antibiotics or immunosuppressant, recent respiratory, urinary or skin infections, presence of wounds and recent hospitalization were also included in the questionnaire. Each subject was also asked to fill in details such as job duration, cleaning area, exposure to infectious waste and body fluid and wear of personal protective equipment.

### Statistics

We used Statistical Package for the Social Sciences (SPSS software for Windows, version 17.0) for statistical analyses. Two-sample *z*-tests were used to compare two proportions, while two-sample *t*-tests were performed to compare means between two continuous variables. For analyses of categorical data, Chi-square tests and Fisher’s exact tests were alternatively used according to the sample size. A p-value<0.05 indicated a statistical significant difference. Odds ratios (ORs) were also calculated with 95% confidence intervals (CIs).

## Results and Discussion

### Subjects

A total of 186 (49.5%) among 376 eligible subjects participated in this study, including 111 (64.5%) out of 172 in the hospital group and 75 (36.8%) out of 204 in the non-medical group. Age of subjects ranged from 21 to 77 years, with a mean age of 58.13 years. The male-to-female ratio was 0.22:1. Of the study subjects, 111 (60%) were hospital janitors while the remaining 75 (40%) were non-medical janitors.

### Nasal carriage of *S*. *aureus* and MRSA among hospital and non-medical janitors

Overall, *S*. *aureus* was found to be colonized in 17 (15.3%) hospital janitors and 10 (13.3%) non-medical janitors, while MRSA in 4 (3.6%) hospital janitors and 1 (1.3%) non-medical janitors. There was no statistically significant difference in the nasal carriage rate of *S*. *aureus* between hospital janitors and non-medical janitors (p = 0.707), as well as that of MRSA between the two groups (p = 0.65). (Table 1)

**Table 1:** Nasal carriage of *S*. *aureus* and methicillin-resistant *S*. *aureus* (MRSA) among janitors working in hospitals and non-medical institutions.

	Hospital janitors	Non-medical janitors	Total	Odds ratio (95% CI)	*p*-value
Eligible subjects	172	204	376		
Participants No.	111	75	186		
*S*. *aureus*	17 (15.3%)	10 (13.3%)	27 (14.5%)	0.851 (0.366–1.976)	0.707
MRSA	4 (3.6%)	1 (1.3%)	5 (2.7%)	0.361 (0.040–3.299)	0.650
Mean age (years)	60.0	56.3	58.1		0.024
Male gender, %	17.1%	18.7%	17.7%		0.779
Mean duration of work (years)	8.73	6.86	7.97		0.053

### Risk factor assessment for MRSA and *S*. *aureus* carriage among hospital and non-medical janitors

Potential risk factors for the acquisition of MRSA in all janitors are shown in Table 2 and those for the acquisition of *S*. *aureus* in hospital janitors in Table 3. In terms of potential risk factors for MRSA acquisition, including age, gender, education level, culture, habits, underlying diseases, drug history and work experience, there was no statistically significant difference between those who carried MRSA and those who did not. However, janitors without allergic rhinitis (p = 0.048), working in hospitals for 6 years or more (p = 0.014) and in charge of cleaning laboratories (p = 0.018) were found to be significantly associated with nasal *S*. *aureus* colonization.

**Table 2:** Association of *S*. *aureus* and methicillin-resistant *S*. *aureus* (MRSA) colonization with demographics, work experience and job details of hospital janitors and non-medical janitors.

	Nasal carriage of MRSA			Nasal carriage of *S*. *aureus*		
	No. (%) of subjects		*p*-value^a^	No. (%) of subjects		*p*-value^a^
	Carriers (n = 5) No. (%)	Non-carrier (n = 181) No. (%)		Carriers (n = 27) No. (%)	Non-carrier (n = 159) No. (%)	
**Age group (years)**						
≦60 (n = 99)	1 (20.0)	98 (54.1)	0.187	13 (48.1)	86 (54.1)	0.567
>60 (n = 87)	4 (80.0)	83 (45.9)		14 (51.9)	73 (45.9)	
**Gender**						
Male (n = 33)	1 (20.0)	32 (17.7)	1.000	5 (18.5)	28 (17.6)	1.000
Female (n = 153)	4 (80.0)	149 (82.3)		22 (81.5)	131 (82.4)	
**Education attainment**						
Elementary school or less (n = 90)	3 (60.0)	87 (48.1)	0.674	14 (51.9)	76 (47.8)	0.699
Junior high school or more (n = 96)	2 (40.0)	94 (51.9)		13 (48.1)	83 (52.2)	
**Culture/ Nationality**						
Islanders (n = 149)	4 (80.0)	145 (80.1)	0.853	24 (88.9)	125 (78.6)	0.388
Mainlanders (n = 11)	0	11 (6.1)		0	11 (6.9)	
Aborigines (n = 5)	0	5 (2.8)		0	5 (3.1)	
Foreign nationality (n = 21)	1 (20.0)	20 (11.0)		3 (11.1)	18 (11.3)	
**Habit**						
Smoking history (n = 24)	0	24 (13.3)	1.000	1 (3.7)	23 (14.5)	0.210
Alcohol history (n = 8)	0	8 (4.4)	1.000	1 (3.7)	7 (4.4)	1.000
Betel nut history (n = 3)	0	3 (1.7)	1.000	0	3 (1.9)	1.000
**Underlying diseases**						
Hypertension (n = 43)	3 (60.0)	40 (22.0)	0.082	6 (22.2)	37 (23.3)	0.905
Diabetes mellitus (n = 20)	4 (80.0)	16 (8.8)	0.438	4 (14.8)	16 (10.1)	0.500
Cardiovascular disease (n = 15)	0	15 (8.3)	1.000	3 (11.1)	12 (7.5)	0.461
Liver disease (n = 8)	0	8 (4.4)	1.000	1 (3.7)	7 (4.4)	1.000
Renal disease (n = 2)	0	2 (1.1)	1.000	0	2 (1.3)	1.000
Respiratory disease (n = 7)	0	7 (3.9)	1.000	0	7 (4.4)	0.596
Sinusitis (n = 5)	0	5 (2.8)	1.000	0	5 (3.1)	1.000
Allergic rhinitis (n = 22)	0	22 (12.2)	1.000	0	22 (13.8)	0.048
**Drug history**						
Steroid (n = 7)	0	7 (3.9)	1.000	0	7 (4.4)	0.596
Antibiotics (n = 2)	0	2 (1.1)	1.000	0	2 (1.3)	1.000
**History within recent 3 months**						
Respiratory disease (n = 25)	0	25 (13.8)	1.000	3 (11.1)	22 (13.8)	1.000
Skin infection (n = 12)	0	12 (6.6)	1.000	1 (3.7)	11 (6.9)	1.000
Current unhealed wound (n = 14)	0	14 (7.7)	1.000	1 (3.7)	13 (8.2)	0.697
Hospitalization (n = 1)	0	1 (0.55)	1.000	0	1 (0.6)	1.000
**Currently living with children** (n = 34)	1	33 (18.2)	1.000	6 (22.2)	28 (17.6)	0.592
**Total job duration as a janitor**						
≦8 years (n = 103)	3 (60.0)	100 (56.9)	1.000	12 (44.4)	91 (57.2)	0.216
>8 years (n = 83)	2 (40.0)	81 (44.8)		15 (55.6)	68 (42.8)	

^a^ Fisher’s exact test instead of Pearson’s chi-square test was performed when any expected count was less than 5 by statistical analysis.

**Table 3:** Association of *S*. *aureus* colonization with demographics, work experience and job details of janitors working in hospitals.

	nasal carriage of *S*. *aureus*				
	No. (%) of subjects		odds ratio	95% CI	*p*-value^a^
	Carriers (n = 17)	Non-carrier (n = 94)			
**Job duration as a janitor in hospitals**					
≦6 years (n = 44)	2 (11.8%)	42 (44.7%)	6.058	1.311–27.987	0.014
> 6 years (n = 67)	15 (88.2%)	52 (55.3%)			
**Cleaning area**					
general ward (n = 54)	6 (35.3%)	48 (51.1%)	0.523	0.179–1.530	0.231
ICU, ER, OR (n = 27)	6 (35.3%)	21 (22.3%)	1.896	0.627–5.735	0.355
OPD, examination room (n = 34)	6 (35.3%)	28 (29.8%)	1.286	0.433–3.818	0.650
laboratory (n = 8)	4 (23.5%)	4 (4.3%)	6.923	1.540–31.119	0.018
office (n = 13)	4 (23.5%)	9 (9.6%)	2.906	0.781–10.819	0.112
rest room (n = 76)	12 (70.6%)	64 (68.1%)	1.125	0.363–3.482	0.838
food court (n = 1)	0 (0.0%)	1 (1.1%)			1.000
hallway (n = 33)	5 (29.4%)	28 (29.8%)	0.982	0.316–3.050	0.975
elevator/ stairs (n = 4)	0 (0.0%)	4 (4.3%)			1.000
**Infectious waste disposal** (n = 98)	16 (94.1%)	82 (87.2%)	2.341	0.284–19.297	0.687
**Exposure to body fluids** (n = 96)	14 (82.4%)	82 (87.2%)	0.683	0.171–2.732	0.699
**Personal protection equipment**					
mask (n = 108)	17 (100.0%)	91 (96.8%)			1.000
gloves (n = 110)	17 (100.0%)	93 (98.9%)			1.000
apron (n = 92)	15 (88.2%)	77 (81.9%)	1.656	0.346–7.928	0.732
gown (n = 4)	1 (5.9%)	3 (3.2%)	1.896	0.185–19.382	0.491
**Types of PPE (mask, gloves, apron, gown)**					
≦2 types (n = 20)	1 (5.9%)	19 (20.2%)	4.053	0.505–32.512	0.300
> 2 types (n = 91)	16 (94.1%)	75 (79.8%)			
**Frequency of changing PPE**					
≦1 per day (n = 80)	12 (70.6%)	68 (72.3%)	1.090	0.350–3.397	1.000
> 1 per day (n = 31)	5 (29.4%)	26 (27.7%)			
**Changing PPE when cleaning different area** (n = 36)	6 (35.3%)	30 (31.9%)	1.164	0.393–3.444	0.784
**Hand hygiene agent**					
water (n = 1)	0 (0.0%)	1 (1.1%)			0.321
soap (n = 74)	14 (82.4%)	60 (63.8%)			
alcohol or iodine (n = 36)	3 (17.6%)	33 (35.1%)			

ICU, intensive care unit; ER, emergency room; OR, operation room; OPD, outpatient department; PPE, personal protection equipment.

^a^ Fisher’s exact test instead of Pearson’s chi-square test was performed when any expected count was less than 5 by statistical analysis.

### Molecular characteristics and antibiotic susceptibility of MRSA and MSSA isolates

**Fig 1:**
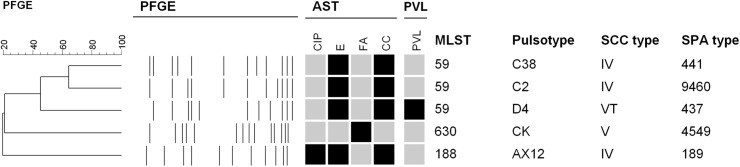
Molecular characteristics of methicillin-resistant *Staphylococcus aureus* isolates from five janitors. All five isolates were resistant to penicillin, and susceptible to vancomycin, teicoplanin, linezolid, doxycycline and trimethoprim-sulfamethoxazole. Antimicrobial susceptibility tests (AST): black indicates resistance, and grey indicates susceptibility. CIP: ciprofloxacin; E: erythromycin; FA: fusidic acid; CC: clindamycin; PFGE: pulsed-field gel electrophoresis; PVL: black indicates that Pantone-Valentine leucocidin genes were detected; SCC: staphylococcal chromosome cassette; MLST: multilocus sequence typing.

Fig 1 reveals the detailed molecular characteristics of the five MRSA isolates from janitors. Four PFGE patterns were identified. All five isolates carried SCC*mec* IV or V. PVL genes were detected in one isolate carrying SCC*mec* V_T_. Three isolates belonged to sequence type (ST) 59, the common sequence type of CA-MRSA in Taiwan. Two other less common sequence types were ST188/ *spa* t189 and ST630/ *spa* t4549 (single locus variant of ST8, ST239), the latter being the type of the only MRSA isolate from non-medical janitors. All five isolates were resistant to penicillin and were susceptible to vancomycin, teicoplanin, linezolid, doxycycline and trimethoprim-sulfamethoxazole. Only one isolate was susceptible to erythromycin and clindamycin while the others were resistant to both antibiotics.

**Fig 2:**
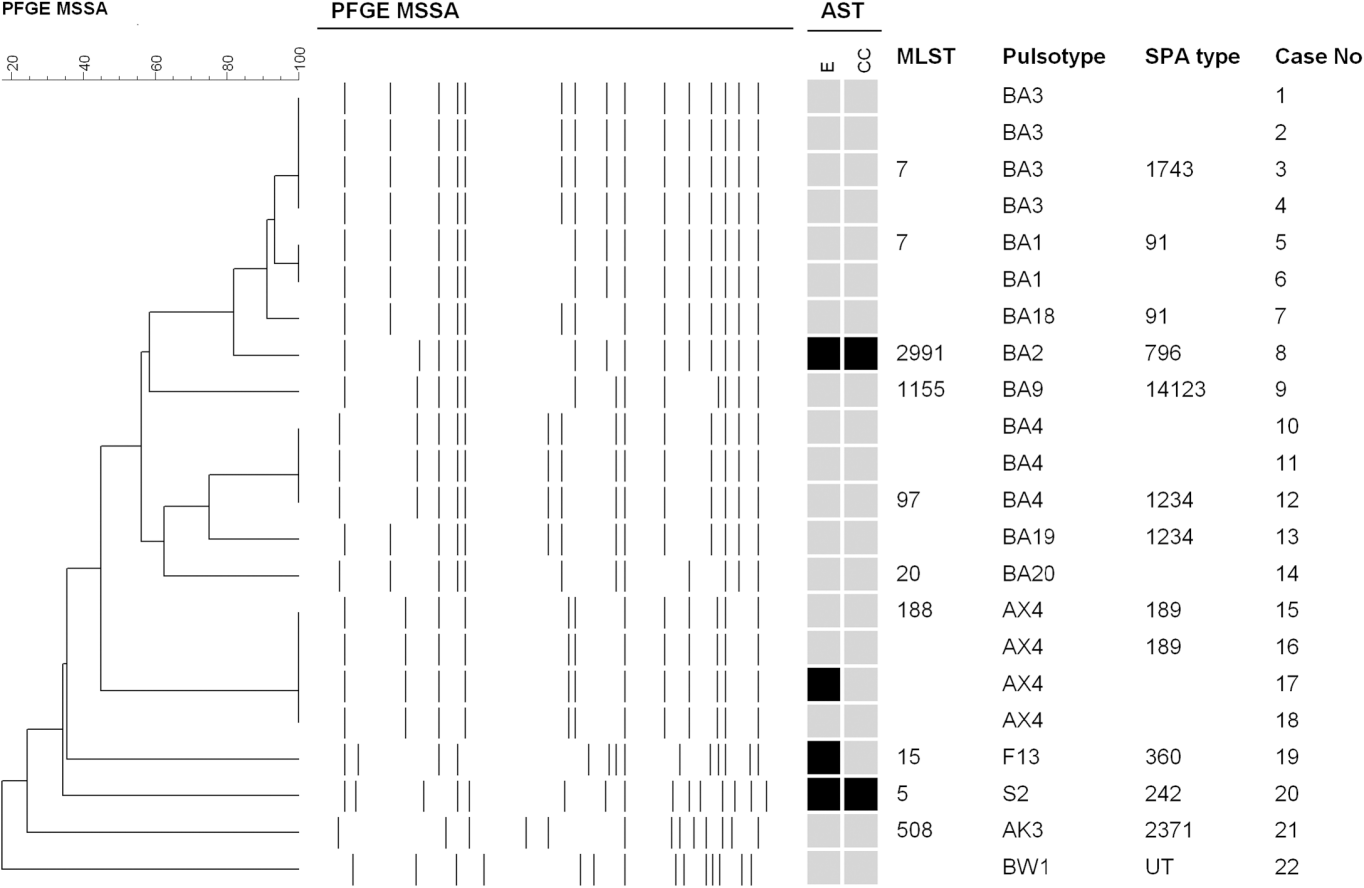
Molecular characteristics of methicillin-sensitive *Staphylococcus aureus* isolates from 22 janitors. All 22 isolates were resistant to penicillin, and susceptible to vancomycin, teicoplanin, linezolid, doxycycline, trimethoprim-sulfamethoxazole, ciprofloxacin and fusidic acid. Antimicrobial susceptibility tests (AST): black indicates resistance, and grey indicates susceptibility. E: erythromycin; CC: clindamycin; PFGE: pulsed-field gel electrophoresis; SCC: staphylococcal chromosome cassette; MLST: multilocus sequence typing.

Of the 22 MSSA isolates, six PFGE patterns with a major type (type BA, 64%) were identified (Fig 2). The 14 MSSA isolates with PFGE type BA were shared by five STs, including ST7, 97, and five spa types, respectively. All four (18%) MSSA strains of PFGE type AX belonged to ST188/ spa t189. All but one MSSA isolates were resistant to penicillin and all of them were susceptible to vancomycin, teicoplanin, linezolid, doxycycline, fusidic acid, ciprofloxacin and trimethoprim-sulfamethoxazole. All but four isolate were also susceptible to erythromycin and clindamycin.

The present study was a preliminary study on epidemiology of *S*. *aureus* and MRSA among janitors working in hospitals in Taiwan and indicated that the nasal carriage rate of MRSA among janitors working in hospitals (3.6%) was comparable to that among those working in non-medical institutions (1.3%). The rate was also comparable to that for healthy adults in community settings (3.8%) in Taiwan [19]. In addition, all five MRSA isolates in this study, either from hospital group or from non-medical group, carried either type IV or V SCC, suggesting community origin. In previous reports from Taiwan [9, 16], both HA-MRSA, though fewer, and CA-MRSA isolates were identified from HCWs. In addition to the dissemination of CA-MRSA in Taiwan, one possible reason why the MRSA isolates from janitors in hospital settings were not HA-MRSA is that janitors working in hospitals are well-equipped with suitable personal protective equipments, as they have a high rate of wearing gloves (99.1%) and surgical masks (97.3%) while working. Moreover, under the supervision of foremen, janitors working in hospitals are requested to strictly follow the correct procedures of medical waste disposal and hand hygiene. This may also partly explain the reason why janitors working in hospital, though exposed to the medical environment, did not have a higher nasal MRSA colonization rate than that for general population.

Due to the relatively small sample size, we failed to identify any risk factor of colonizing MRSA among janitors in Taiwan. Nevertheless, among the janitors working in hospitals, we found those working in hospitals for 6 years or more (15/67, 22%) and those cleaning laboratories (4/8, 50%) were significantly associated with *S*. *aureus* colonization. The finding that working in the laboratories may lead to a higher rate of *S*. *aureus* colonization had been reported previously [20]. This raises our concern of the health welfare of janitors working in the laboratories and their families outside the medical environment. In addition, Schmidlin et al also reported that laboratory staff with *S*. *aureus* colonization might contaminate the surfaces of working environment and consequently spread the pathogen [21]. All these findings suggest that janitors working in the laboratories should be more meticulous and strictly follow the infection control measures to prevent acquisition of a potential pathogen from or contaminating the laboratory surfaces.

Unexpectedly, the present study indicated that janitors with allergic rhinitis were less vulnerable to be colonized with *S*. *aureus*, in contradiction to the findings observed in previous reports [22]. Since the sample size was small in this study, further studies are therefore needed to clarify the issue whether there is an association between allergic rhinitis and the carriage of *S*. *aureus*.

In the present study, we also found that nearly two-thirds of MSSA isolates shared a common PFGE type, namely type BA (belonged to ST7, 97 and others), a sequence type possibly prevailing in hospitals as well as in the community. This PFGE type BA was also predominant in a cluster of MSSA colonization in the nursery of Chang Gung Memorial Hospital at Linkou and in a survey of *S*. *aureus* colonization among neonatal intensive care units in Taiwan [23, 24]. The clinical significance and impact of this MSSA strain in Taiwan needs further studies since there have been scanty reports regarding molecular epidemiology of MSSA in Taiwan.

The main limitation of the current study is a low participation rate of eligible janitors. Only nearly two-thirds of the eligible subjects in the hospital group participated in this study, and the rate was even lower in the non-medical group (36.8%). The case number size was hence smaller than expected, leading to a reduced statistical power. On the other hand, only single cross-sectional sampling was obtained, hence more evidence was needed for disclosure of long term carriage status of both MRSA and MSSA. Despite of its limitations, this study can still provide an epidemiologic feature of *S*. *aureus* carriage in this population and thus implement better infection control measures.

## Supporting Information

S1 Table Drug resistance profile and molecular characteristics of methicillin-sensitive *S*. *aureus* isolates. (DOCX)
