# Supplementary material for: Nasal Staphylococcus aureus and Methicillin-Resistant S. aureus Carriage among Janitors Working in Hospitals in Northern Taiwan
Source: PLoS One. 2015 Sep 25;10(9):e0138971. doi: 10.1371/journal.pone.0138971 (PMC4583260; doi:10.1371/journal.pone.0138971)
Supplement: S1 Table — (DOCX) [file pone.0138971.s001.docx]

# Supporting Information

| **S1 Table. Drug resistance profile and molecular characteristics of methicillin-sensitive *S. aureus* (MSSA) isolates** | | | | | | | | | | | | | | |
| --- | --- | --- | --- | --- | --- | --- | --- | --- | --- | --- | --- | --- | --- | --- |
| **Institution** | **No** | **VA** | **TEC** | **LZD** | **D** | **P** | **CIP** | **E** | **FA** | **CC** | **SXT** | **Pulsotype** | **MLST** | **SPA type** |
| **Hospital** | 1 | S | S | S | S | R | S | S | S | S | S | AX4 | 188 | 189 |
|  | 2 | S | S | S | S | R | S | R | S | S | S | AX4 |  |  |
|  | 3 | S | S | S | S | R | S | S | S | S | S | AX4 |  |  |
|  | 4 | S | S | S | S | R | S | S | S | S | S | BA1 | 7 | 91 |
|  | 5 | S | S | S | S | R | S | S | S | S | S | BA18 |  | 91 |
|  | 6 | S | S | S | S | R | S | S | S | S | S | BA19 |  | 1234 |
|  | 7 | S | S | S | S | R | S | S | S | S | S | BA20 | 20 |  |
|  | 8 | S | S | S | S | R | S | S | S | S | S | BA3 |  |  |
|  | 9 | S | S | S | I | R | I | S | S | S | S | BA3 |  |  |
|  | 10 | S | S | S | I | R | S | S | S | S | S | BA3 | 7 | 1743 |
|  | 11 | S | S | S | S | R | S | S | S | S | S | BA4 |  |  |
|  | 12 | S | S | S | I | R | S | S | S | S | S | BW1 |  | UT |
|  | 13 | S | S | S | S | R | S | R | S | R | I | S2 | 5 | 242 |
| **Non-hospital** | 14 | S | S | S | S | S | S | S | S | S | S | AK3 | 508 | 2371 |
|  | 15 | S | S | S | S | R | S | S | S | S | S | AX4 |  | 189 |
|  | 16 | S | S | S | S | R | S | S | S | S | S | BA1 |  |  |
|  | 17 | S | S | S | S | R | S | R | S | R | S | BA2 | 2991 | 796 |
|  | 18 | S | S | S | S | R | S | S | S | S | S | BA3 |  |  |
|  | 19 | S | S | S | S | R | S | S | S | S | S | BA4 |  |  |
|  | 20 | S | S | S | S | R | S | S | S | S | S | BA4 | 97 | 1234 |
|  | 21 | S | S | S | S | R | S | S | S | S | S | BA9 | 1155 | 14123 |
|  | 22 | S | S | S | S | R | S | R | S | S | S | F13 | 15 | 360 |

VA: vancomycin, TEC: Teicoplanin, LZD: Linezolid, D: Doxycycline, P: Penicillin, CIP: Ciprofloxacin, E: Erythromycin, FA: Fusidic acid, CC: clindamycin, SXT: Trimethoprim-sulfamethoxazole, MLST: multilocus sequence typing
